# Use of Distributed Temperature Sensing Technology to Characterize Fire Behavior

**DOI:** 10.3390/s16101712

**Published:** 2016-10-17

**Authors:** Douglas Cram, Christine E. Hatch, Scott Tyler, Carlos Ochoa

**Affiliations:** 1 Extension Animal Sciences and Natural Resources, New Mexico State University, Las Cruces, NM 88003, USA; 2 Department of Geosciences, University of Massachusetts, Amherst, MA 01003, USA; chatch@geo.umass.edu; 3 Geological Sciences and Engineering, University of Nevada, Reno, NV 89557, USA; styler@unr.edu; 4 Department of Animal and Rangeland Sciences, Oregon State University, Corvallis, OR 97331, USA; Carlos.Ochoa@oregonstate.edu

**Keywords:** fiber-optic, FO-DTS, prescribed fire, rangeland, spatial mapping

## Abstract

We evaluated the potential of a fiber optic cable connected to distributed temperature sensing (DTS) technology to withstand wildland fire conditions and quantify fire behavior parameters. We used a custom-made ‘fire cable’ consisting of three optical fibers coated with three different materials—acrylate, copper and polyimide. The 150-m cable was deployed in grasslands and burned in three prescribed fires. The DTS system recorded fire cable output every three seconds and integrated temperatures every 50.6 cm. Results indicated the fire cable was physically capable of withstanding repeated rugged use. Fiber coating materials withstood temperatures up to 422 °C. Changes in fiber attenuation following fire were near zero (−0.81 to 0.12 dB/km) indicating essentially no change in light gain or loss as a function of distance or fire intensity over the length of the fire cable. Results indicated fire cable and DTS technology have potential to quantify fire environment parameters such as heat duration and rate of spread but additional experimentation and analysis are required to determine efficacy and response times. This study adds understanding of DTS and fire cable technology as a potential new method for characterizing fire behavior parameters at greater temporal and spatial scales.

## 1. Introduction

Fire science, effects, and modeling research require measures of fire behavior parameters such as fireline intensity, heat duration, and rates of spread [1,2]. Quantifying these parameters is challenging in the laboratory and notoriously difficult in the field where instrument limitations, variations in fuel characteristics, and the turbulent nature of fluid dynamics and combustion lead to drastic variations over time and space. Options for quantifying these parameters include remotely sensed data from instruments mounted on aircraft or satellites with large pixel footprints or ground-based sensors within or above the flames [2]. Spatial limitations are a significant shortcoming of current ground-based instruments when used to quantify fire behavior parameters because they operate at an individual point scale (e.g., thermocouples) [3,4]. Filling the gap in the spatial domain as well as the temporal scale can be critical to improving our understanding of fire behavior in wildland fuels.

We tested a custom built fiber-optic ‘fire cable’ using distributed temperature sensing (DTS) technology under wildland fire conditions. Distributed temperature sensing technology has become a widely-used environmental monitoring tool in recent years ranging from applications in hydrology [5], atmospheric science [6] and oceanography [7]. Advantages of DTS technology include the opportunity to record data at a high level of precision and at high spatial and temporal frequencies in air, water, soil or other media. Distributed temperature sensing technology is also widely used for industrial [8] and transportation (e.g., tunnel) fire detection [9]. In fire detection applications, DTS is used primarily to indicate presence or absence of fire rather than continuously monitoring fire behavior parameters. Distributed temperature sensing technology has not been used in natural settings for fire characterization nor has it been used for prescribed fire applications.

Distributed temperature sensing technology is based on sending (launching) a light pulse down an optical fiber and analyzing the return signal of backscattered Raman-spectra light at specific frequencies that are either sensitive (anti-Stokes) or not sensitive (Stokes) to changes in temperature. Photons returning within specific frequency bands are recorded in timed ‘bins’ which, based on the speed of light, represent a discrete length of cable a known distance from the detector. After calibration to locations of known temperature along the cable, temperatures can be resolved for the entire length of the cable at the desired time interval. Typical commercial DTS systems provide an integrated temperature every 1–2 m of fiber at frequencies from 0.01 to 1 Hz. We conservatively estimated and reported temperature resolutions to ~0.1 °C as achieved in the field.

Distributed temperature sensing systems are capable of measuring temperatures as high as 600 °C and since the optical fiber is glass, high temperatures can be tolerated. However, repeated and prolonged exposure to high temperatures (i.e., >400 °C) promotes or accelerates ‘fiber darkening’ a product of hydrogen contamination, especially in hydrogen-saturated environments such as petrochemical applications [10,11]. The presence of free hydrogen and other weathering products results in greater adsorption of incident light over time, resulting in less light transmitted, and less light returned to detectors. Typical optical fiber used for telecommunication is designed to have insignificant darkening at temperatures between 60 °C and 85 °C over the life of operation (e.g., tens of years). At temperatures above this design threshold, darkening may occur more rapidly, resulting in time-dependent light transmission/scattering behavior. To overcome this issue, fibers can be coated with a variety of materials including gold [12] and then surrounded by a hydrogen scavenging gel. In this work, we tested several different fiber coatings to determine how they performed following multiple prescribed fires.

Additional fiber challenges related to wildland conditions are as follows: the optical fiber is fragile (e.g., typical diameter is 125 microns) and must be protected from strain and abrasion. Typically, strength elements are designed to mitigate longitudinal strain, stainless steel capillary tubes protect the fiber from radial strain, and plastic jacketing is used to protect the fiber optic cable from ultraviolet radiation. In this work, we consider the fiber-optic fire cable to include optical fibers (i.e., three fibers), coating material on the optical fibers (three different materials were used), and strength elements (the cable used in this study did not have a protective jacket). The objective of this study was to evaluate the potential of a fiber optic cable connected to DTS technology to withstand the fire environment (i.e., exposure to repeated combustion temperatures and deployment abuse) and quantify fire behavior parameters such as temperature, heat duration and rate of spread.

## 2. Experimental Section

### 2.1. Study Site

Our experiment site was located on the Corona Range and Livestock Research Center, Torrance County, NM, USA (UTM 13 S 463655 E 3793075 N) at 1870 m above sea level. The ranch is operated by New Mexico State University for range and animal science research and teaching purposes. The burn site vegetation and fuel was characterized as short-grass blue grama (*Bouteloua gracilis*) grassland with little to no shrubs outside of an occasional cholla cactus (*Cylindropuntia imbricata*). Average annual precipitation between 1990 and 2007 was 330 mm, with most of the rain falling between July and September. Average daily maximum air temperature is 10 °C during winter and 29 °C in summer. Average daily minimum air temperature is −3.9 °C in winter and 13.9 °C in summer [13].

### 2.2. Study Design

**Figure 1:**
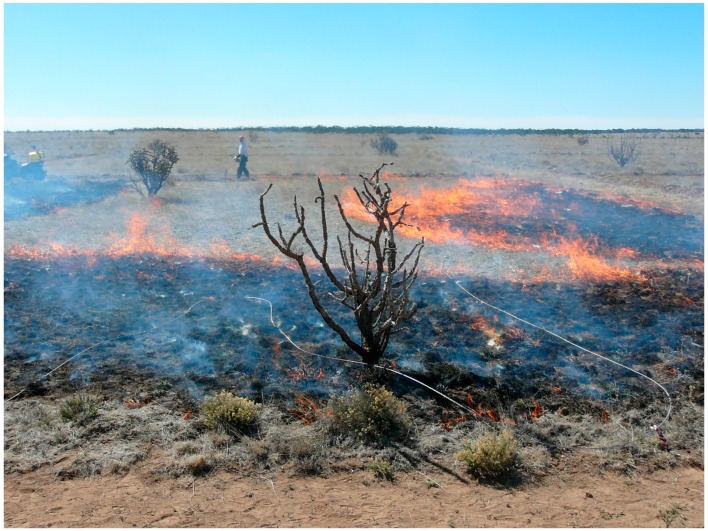
Photograph of prescribed fire (plot 1) at Corona Range and Livestock Research Center, Torrance County, New Mexico showing backfire (visible on the left), flank fire (foreground) and head fire (moving from right to left). The ‘fire cable’ is visible on the ground.

Three prescribed fires were implemented to test the applicability of the DTS technology to measuring fire parameters such as duration (i.e., residence heat time), rate of spread, and temperature. The prescribed burns were conducted on three different 20 by 27 m plots on 15 March 2012. Vegetation was senescent at the time of burn. Herbaceous fuel loading for burn plot one was 733 kg·ha^−1^ (136 SE), and 706 kg·ha^−1^ (66 SE) for burn plot two. No fuel loading was recorded for burn plot three. The size of the rectangular burn plots was established by a prior prescribed fire study at the same location [14]. A 6.7-m firebreak was installed on all four sides of the plots using a road grader. The three burns were ignited at 11:20, 12:56, and 13:52 h, respectively, with each burn lasting about 8 min. Following the burn, the fire cable was moved to a new plot. Each ignition was started with a backfire, followed by a flank fire, and finished with a head fire (Figure 1). A head fire is defined as a fire spreading with the wind. A back fire is defined as a fire spreading against the wind. A flank fire is defined fire spreading perpendicular to the wind.

### 2.3. DTS Technology

**Figure 2:**
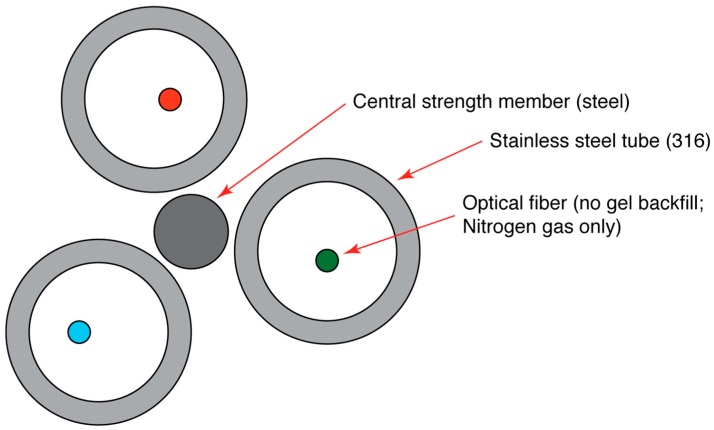
Cross-section schematic of custom-made fiber optic ‘fire cable’. Fire cable consisted of three hollow stainless steel capillary tubes twisted together around a central strength element. Each tube contained one 50/125-μm multi-mode optical fiber. Each fiber had a different coating: copper, polyimide and acrylate. No hydrogen scavenging gel was used for this cable. As such, contrary to the figure illustration, it is likely the optical fibers were in contact with the stainless steel wall of the capillary tubes for much of the length of the cable. Nitrogen gas was pumped through the stainless steel tubes during prescribed fires. Fire cable was constructed by AFL Telecommunications.

Standard telecommunication fiber-optic cables are not designed to withstand long periods at combustion temperatures generated as a result of burning wildland fuels. In order to test the feasibility of using DTS technology for wildland fire applications, a 150-m specialized fiber-optic fire cable was custom made (Figure 2). The fire cable consisted of three optical fibers with three different coatings ranging from a low-cost coating typical of telecommunication applications to specialty materials to protect the optical fibers. Each fiber was armored in a stainless steel capillary tube and backfilled with inert gas. Optical glass is sufficiently heat resistant, so standard multi-mode 50-μm core and 125-μm cladding fiber was used. Typical acrylate buffer coating used on most optical fibers is rated to withstand only 120 °C. We included a fiber coated with acrylate to test its performance in an extreme environment. In addition, we included two more specialized fibers, each coated with a different heat-resistant material: copper and polyimide. Each fiber was encased in an individual, rigid stainless steel tube and overstuffed (i.e., the fiber length is ~0.1% longer than the steel tube length) to accommodate bending. Typically these tubes would be backfilled with a hydrophobic gel to eliminate water, but since the gels have been known to expand and flow out of the tubes when heated, the tubes were not filled with gel. Instead, during prescribed fire operations a small amount of dry nitrogen gas was continuously pumped through all three stainless-steel tubes to protect the fibers, prevent air or water from entering, and reduce condensation from occurring inside the tubes. Three stainless-steel tubes were braided together around a single steel strength member to form the finished armored cable. The resulting braided bundle had a finished diameter of 9 mm. Typically, cables are encased in UV-resistant or other protective plastic, but to maximize the thermal response, we left the metal bundle without additional coating. Since the predominant mode of heat transport for sensing will be conductive, we expect the dominantly metal cable bundle to respond rapidly (seconds) to changes in ambient temperature. It should also be noted that fibers are likely in contact with the stainless steel walls of the capillary tube due to their helical arrangement inside the tube (generated by ~1% over-stuffing) and due to the absence of hydrophobic gel.

In order to evaluate the physical performance of each fiber coating in the fire cable we compared the signal strength and light propagation of one Raman frequency (i.e., Stokes) before and after the prescribed burns. The incident laser light used by our DTS was 1040 nm, which is Raman backscattered into Stokes and anti-Stokes wavelengths of 1080 nm and 1000 nm, respectively. We used the Stokes backscattered light for this analysis (i.e., 1080 nm). Because there is variability between light pulses we averaged 50 traces before the cable was subjected to the burn and 50 traces after the burn. The cable was not disconnected or impinged between trace sampling. As a result, we assumed the only change between these two averages of light intensity was due to fire effects on the cable. For each meter along the cable, we calculated the amplitude of light intensity lost (in decibels/km) since its ‘launch’ into the fiber from the DTS unit for Stokes frequency. We then subtracted the averages (before minus after) to assess change due to heating on the cable for all three fibers.

### 2.4. Field Data Collection

**Figure 3:**
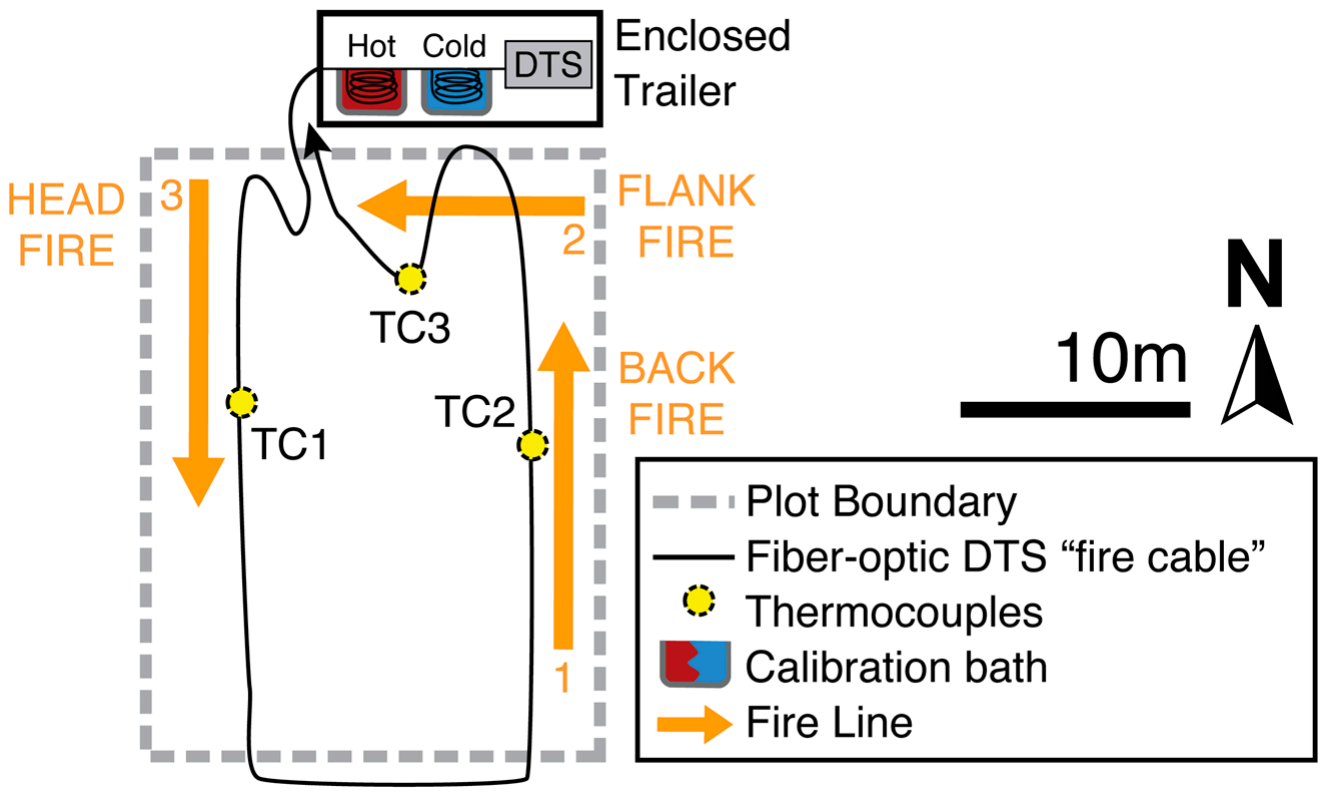
Schematic layout of 150-m fiber-optic ‘fire cable’ in experimental burn plots. This layout was repeated in plots 1, 2, and 3. Each grassland plot was 20 by 27 m. The fire cable runs from the distributed temperature sensing (DTS) unit into a cold calibration bath, into a hot calibration bath, out into the plot and back through both baths. The DTS unit and baths were housed in a protective trailer. The fire cable runs in two roughly linear transects approximately 8 m apart. Ignition order was a follows: back fire, flank fire, head fire.

We deployed the fire cable within each burn unit and arranged it to maximize spatial coverage of the experimental plots. Each optical fiber was connected to one channel of an Ultima SR (Silixa Ltd., Hertfordshire, UK) DTS unit housed inside a closed canopy (2.1 by 4.2 m) trailer for protection. We used a portable generator to provide power for the electronic equipment including the DTS unit. Within the trailer, two 75 L plastic coolers were filled with 37 L of warm water and cold water plus ice, respectively to create a ‘warm’ or ambient bath and a ‘cold’ bath used for DTS calibration purposes. While the DTS unit we used had a default calibration, we opted to preform our own calibration test to ensure we collected accurate data that related to actual temperatures. Four eight-meter coils of the fire cable were placed in the warm and cold baths (i.e., two coils at each end of the fire cable). The first and last meter of calibration coil data were discarded, leaving six meters of cable for calibration at each end of the cable in each bath. The Ultima DTS has a minimum sample resolution of 12.5 cm. For this installation, the sampling resolution was increased to 25.3 cm to improve temperature resolution. Typically, the ability to resolve differences in temperature along the fiber (spatial resolution) is approximately twice the sampling resolution. However, for this experiment, we calculated spatial resolution closer to 1 m [15]. Platinum-resistance thermometers (PT100) rated to 0.1 °C accuracy were placed in each bath and temperatures were recorded by the DTS unit at the same three-second intervals as DTS data from the fibers. In addition, independent measurements of fire temperature were obtained using three, type-K (Chromel-Alumel), thermocouples that were deployed during each of the three burn trials. Three thermocouples were positioned within 0.2 cm of the fire cable at three known locations (i.e., 55, 100, and 123 m along fire cable) (Figure 3). These locations corresponded with head, back, and flank fire conditions during burn. Head, back and flank fires were as follows along the fire cable: 30–73 m, 85–112 m, and 118–130 m, respectively. Thermocouples were connected to a CR10X datalogger (Campbell Scientific Inc., Logan, UT, USA) that was programmed to record maximum temperature, time of occurrence, and average temperature at five-second intervals. Figure 3 shows the placement of the fire cable and thermocouples. To quantify the total amount of thermal energy (*TTE*) measured at a known location on each instrument we calculated the sum of measured energy over time (i.e., the duration of the prescribed fire).

## 3. Results and Discussion

**Figure 4:**
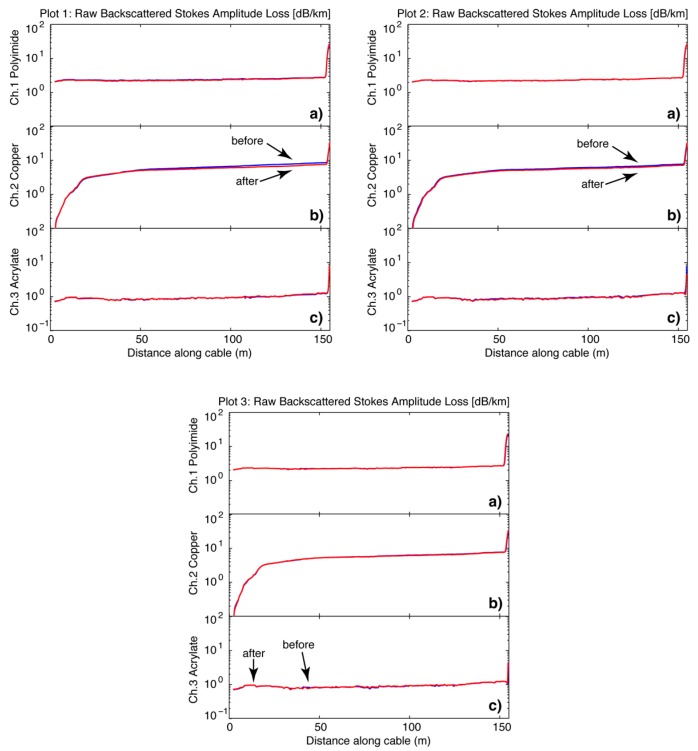
Attenuation (dB/km) between raw DTS data (backscattered Raman Stokes frequency amplitude) before (blue) and after (red) each burn in plot 1, 2, and 3 for (a) Polyimide coated fiber; (b) Copper coated fiber; and (c) Acrylate coated fiber. The first 50 traces of the Stokes signals are averaged together, and the last 50 traces after each burn are averaged together and plotted together. There is little to no change in attenuation after each burn.

Fire cable and DTS technology detected short-grass wildland fire combustion without catastrophic failure during three prescribed fires. This was significant, because to our knowledge, this specific application had not before been tested in wildland fire conditions. Results indicated the fire cable was physically capable of withstanding repeated rugged use. Also of interest was how the three fiber coatings affected attenuation. Changes in optical fiber attenuation following fire were near zero (−0.81 to 0.12 dB/km) for the polyimide and acrylate coated fibers indicating essentially no change in light gain or loss as a function of distance, fire intensity, or maximum temperature reached following three prescribed burns (Table 1). The polyimide and acrylate coated fibers showed a relatively consistent value for attenuation along the entire length of the fire cable (Figure 4). As for the copper coated fiber, while the attenuation signature was logarithmic in shape—which is unusual for optical fibers—the change in attenuation was minor (Figure 4, Table 1). We believe the logarithmic shape of the attenuation signal was an artifact of the manufacturing process (e.g., bend memory) as this condition existed before prescribed burns were conducted. In order to characterize changes in attenuation on this fiber, three sections were selected with different representative slopes for attenuation calculations. Results showed changes from −13.35 to 9.53 dB/km after the prescribed burns (Table 1). We would expect an increase in attenuation if the burn caused any damage to the fiber, yet in two sections of the fiber we observe the opposite. This decreased attenuation may be the result of relaxation of manufacturing effects such as bend memory, but is not likely to have been induced by the burn specifically. Hypothetically, the changing slope along the entire length of the copper fiber could result in increased noise or less accurate temperature measurements further down the cable but this was not supported by the data. The anomalous attenuation curve observed in this particular copper-coated fiber may not be representative of all copper-coated fibers. A microscope examination of the glass fibers following decommission would reveal more about the nature and extent of any possible fiber or coating damage.

**Figure 5:**
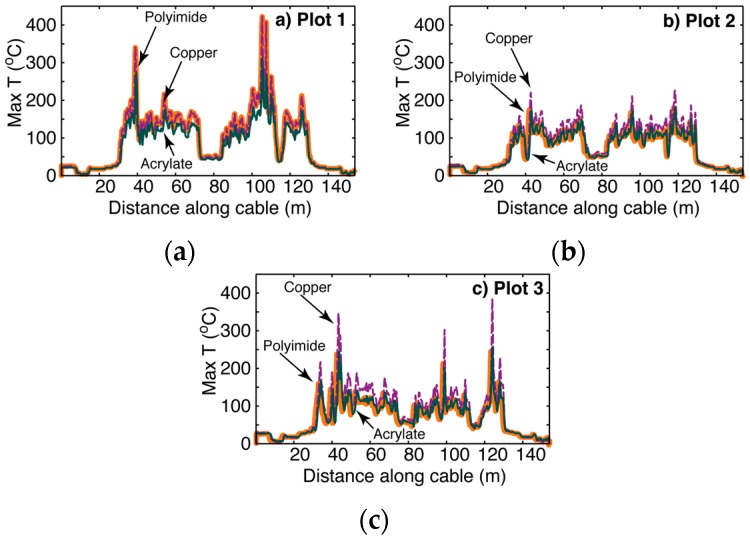
Maximum fire cable temperatures as recorded by DTS technology by distance (m) for polyimide (thicker, orange), copper (dashed purple), and acrylate (green) optical fiber coatings from (**a**) Plot 1 (Max = 421.9 °C on copper); (**b**) Plot 2 (Max = 223.2 °C on copper); and (**c**) Plot 3 (Max = 395.2 °C on copper). Calibration bath locations were as follows: ice baths at 7–15 m and 146–154 m, and ambient temperature baths at 16–24 m and 137–145 m. Head, back and flank fires were as follows: 30–73 m, 85–112 m, and 118–130 m, respectively. Fire cable that was outside the fire line was as follows: 0–29 m, 74–84 m, 113–117, and 131–154 m.

Differences in performance given three different fiber coatings are illustrated in Figure 5, which shows the maximum recorded temperature during the burn vs. distance along the cable for all three burn plots.

**Figure 6:**
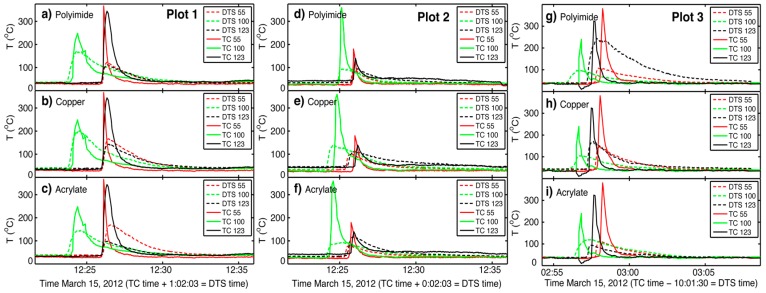
Fire cable temperatures (°C) as recorded by DTS technology (dashed lines) and thermocouples (solid lines) during three prescribed fire at distances of 55, 100, and 123 m along the cable. Data from the three burns (i.e., plots 1, 2, and 3) were recorded from optical fibers with polyimide (**a**,**d**,**g**), copper (**b**,**e**,**h**), and acrylate (**c**,**f**,**i**) coatings. Head (55 m), back (100 m) and flank (123 m) fires were used to burn plots.

As predicted, because of its relatively lower heat capacity, the copper coated fiber recorded greater or near-equal maximum temperatures when compared to the polyimide and acrylate coated fibers at head, back and flank fire locations. The polyimide and acrylate coated fibers recorded similar temperatures across all fire conditions (i.e., head, back, and flank). Absolute peak temperatures recorded by DTS technology were 407, 422 and 310 °C on polyimide, copper and acrylate coated fibers. These peak temperatures were recorded at back fire locations in plot one. Fire cable temperatures derived from DTS technology were considerably lower than thermocouple temperatures at head, back and flank fire locations (i.e., meter 55, 100, and 123) at all three plots (Figure 6, Table 2). The heat capacity of the fire cable may hinder its ability to accurately capture peak temperatures, especially when subjected to the rapid changes associated with head fire conditions (Figure 6).

**Figure 7:**
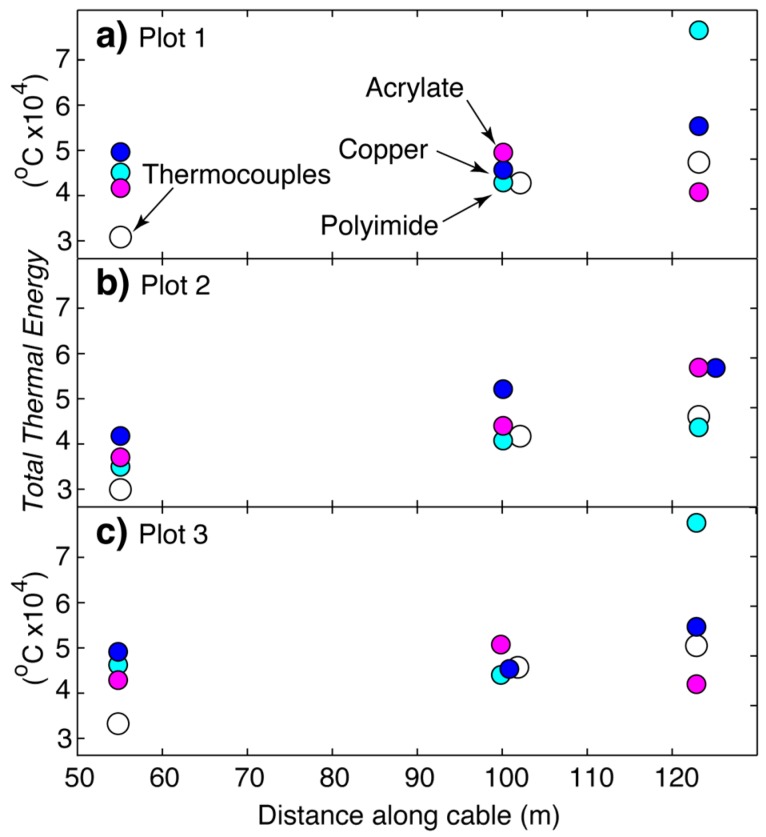
Total thermal energy measured for thermocouple and optical fiber temperatures (°C) from (**a**) Plot 1; (**b**) Plot 2; and (**c**) Plot 3 at distances along the cable of 55 (head fire), 100 (back fire) and 123 (flank fire) m.

In addition, fire cable temperatures across all three fibers on all three burn plots remained greater through time following head, back and flank fire events as compared to thermocouples (Figure 6). Optical fibers measured greater total thermal energy over time as compared to thermocouples at head fire locations (i.e., 55 m) with the copper fiber generally measuring the greatest total energy (Figure 7, Table S1). At back fire locations (i.e., 100 m), total thermal energy was similar between optical fibers and thermocouples, with the copper coated fiber still measuring the greatest *TTE* in most places (Figure 7, Table S1). Total thermal energy was variable at flank fire locations (i.e., 123 m) as compared to head and back fire locations (Figure 7, Table S1). As noted above, the greater thermal mass of the fire cable, including the central steel strength member, helps explain this response.

It should be noted that DTS technology, similar to thermocouples, pyrometers, and calorimeters, cannot record actual flame temperatures, but rather measure its own temperature change during a fire [3,4,16]. Without extensive knowledge of the thermodynamic properties of the fire cable and a model for heat transport [17], these fire cable temperatures are not a surrogate for fire behavior parameters such as energy density or flux density [2]. When measuring temperatures in turbulent flows, time resolution can be an issue due to sub-scale mixing and movement that leads to dramatic fluctuations. If the thermal mass of the fire cable is too great or the time response is too slow these aforementioned fluctuations are smoothed over and the resulting data must be considered an average temperature. However, there is still a need to record these temperatures in the field [15,18,19] but they must be quantified as either peak or averaged temperatures. Bova and Dickinson [4] suggested temperature measuring instruments like thermocouples, and thereby fire cable, can be used to estimate fireline intensity if calibration is performed by comparison with field measurements.

**Figure 8:**
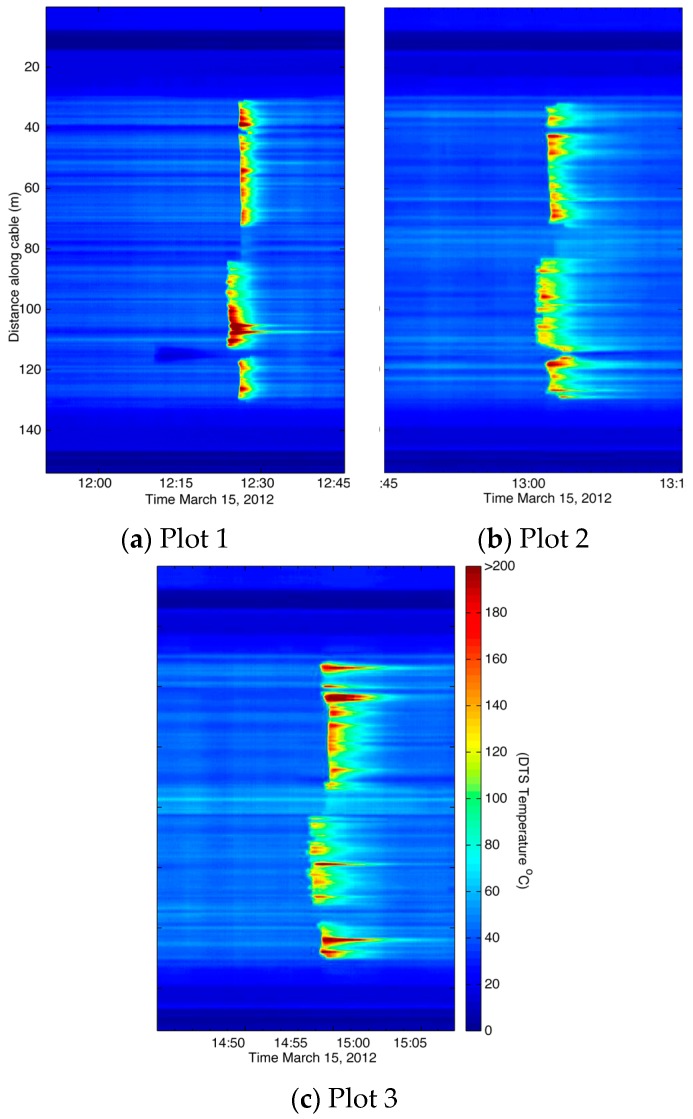
Graph illustrating duration of heat for copper coated optical fiber in (**a**) Plot 1; (**b**) Plot 2; and (**c**) Plot 3. Rate of spread can also be illustrated, however, orientation of cable needs to be perpendicular, and in this trial the fire cable was parallel to fire spread. All temperatures above 200 °C are shaded at the scale maximum; refer to Figure 5 for maximum recorded temperatures along cable. Calibration bath locations were as follows: ice baths at 7–15 m and 146–154 m, and ambient temperature baths at 16–24 m and 137–145 m. Head, back and flank fires were as follows: 30–73 m, 85–112 m, and 118–130 m, respectively. Fire cable that was outside the fire line was as follows: 0–29 m, 74–84 m, 113–117, and 131–154 m.

Potential uses for fire cable and DTS technology in fire science include continuous spatial recording of rate of spread, duration, and fire location (Figure 8). Prior to this technology, the ability to capture the distribution of these parameters across time and space were relatively impractical. Thermocouple technology would allow for spatial mapping of these fire parameters as well, but at notably limited scales (i.e., point sampling) and requiring cumbersome wiring. The ability to map and quantify rate of spread and duration may be useful in fire modeling and effects research, respectively. Additional laboratory and field experimentation are required to gain a better understanding on the efficacy of using fire cable and DTS technology to quantify these parameters.

It is unlikely that a practitioner would construct a fire cable as complex as the one used in this study. Our fire cable was constructed with additional thermal mass for experimentation purposes, but future fire cable iterations may avoid this potentially challenging condition. Future constructions of a fire cable can take into account lessons learned in this study. For example, thermal mass challenges can be mitigated as follows: (1) discard the strengthening member; (2) include only one optical fiber thus requiring only one armoring tube; and (3) utilize a smaller diameter armoring tube. These options would also reduce construction costs. Further, we offer some thoughts on the cost of constructing specialty cables. Often the exterior armoring is the most expensive part ranging from $5–10/m. Individual fiber coatings can also add significant cost. The easily available acrylate coated fiber adds $0.01–0.10/m, polyimide coating adds $0.3–5/m, and copper coating adds $20–30/m. Although the copper coated fiber may outlast the others, the additional cost may not be worth the difference depending on application objectives.

## 4. Conclusions

This study adds to the understanding of using DTS technology for fire science research and establishes a secondary starting point for testing the next generation fire cable at larger spatial scales. The capabilities of this technology may be useful for fire behavior scientists and fire ecologists (Table 3). For example, quantifying fire behavior in heterogeneous fuel beds is difficult, but may be facilitated using this technology. Fiber optic cables act as a single continuous monitoring device that enables representation of fire distribution through spatial domains, something that was practically impossible to achieve with other fire-resistant instruments. Results from this study indicate DTS technology coupled with a fiber-optic fire cable have potential to map and quantify fire parameters such as duration, rate of spread, and fire location at detailed time and space intervals. However, additional laboratory and field testing is required before this technique can be evaluated with regard to these applications.

## Figures and Tables

**Table 1:** Losses in Raman backscattered amplitude (Stokes frequency) following three prescribed burns.

DTS Fiber Coating	Before Burn			After Burn		Change ^b,c^
	Loss (dB)	Length (m) ^a^	Attenuation (dB/km)	Loss (dB)	Attenuation (dB/km)	Attenuation (dB/km)
Polyimide	0.22	100.7	2.1	0.13	1.9	−0.81
Copper	1.49	11.8	126.8	1.60	135.8	9.53
	2.63	33.0	79.7	2.19	69.3	−13.35
	2.96	95.8	30.9	2.11	24.5	−8.78
Acrylate	0.26	121.2	2.2	0.28	2.3	0.12

^a^ Attenuation was calculated before and after burns over a length of cable with representative slope. For the polyamide-coated fiber, loss was calculated from 20.6 to 121.2 m. For the copper-coated fiber where attenuation was changing, three representative sections were chosen from 3.2 to 15.0 m, 18.0 to 51.1 m, and 56.0 to 151.7 m. Acrylate losses were calculated from 20.5 to 141.7 m; ^b^ Calculated as average attenuation (dB/km) of the last 50 traces after burning plot 3 minus average attenuation of the first 50 traces before burn in plot 1; positive values indicate an increase in attenuation; negative values indicate a decrease in attenuation; ^c^ Fibers with consistent attenuation (i.e. relatively constant slope of Stokes intensity vs. distance) have near-zero change in attenuation (i.e., polyimide, acrylate). Large decreases in attenuation are most likely a relaxation of fabrication-induced attenuation, rather than any effect of fiber burning, which would be expected to increase attenuation if damage to fibers occurred.

**Table 2:** Maximum temperature as measured by thermocouple and DTS fire cable during three prescribed burns.

Location and Sensor		T (°C) at 55 m ^a^	T (°C) at 100 m	T (°C) at 123 m	Absolute Maximum T (°C) ^b^	Location (m) ^c^
Plot 1						
Thermocouple		366.7	248.1	344.0		
DTS fiber coating						
	polyimide	122.7	173.1	112.3	307.1	104
	copper	166.1	202.3	98.5	421.9	105
	acrylate	169.1	145.7	143.2	310.1	106
Plot 2						
Thermocouple		184.4	363.2	144.1		
DTS fiber coating						
	polyimide	91.7	97.6	100.1	178.1	118
	copper	105.1	141.7	132.4	223.2	118
	acrylate	82.81	100.8	122.2	178.3	119
Plot 3						
Thermocouple		380.7	239.6	324.0		
DTS fiber coating						
	polyimide	108.9	97.1	243.4	243.4	123
	copper	147.9	105.4	170.6	395.2	124
	acrylate	113.6	121.4	94.0	254.2	125

^a^ Meter mark along fiber-optic cable where maximum temperature was measured by DTS and thermocouple; ^b^ Maximum temperature as measured along entire length of fire cable by DTS; ^c^ Location of maximum temperature as measured along entire length of fire cable by DTS.

**Table 3:** Potential strengths and weaknesses of using fire cable and DTS technology to quantify wildland fire behavior parameters, and a summary of alternative methods.

Fire Behavior Parameter	Definition	Fire Cable and DTS Technology	Alternative Methods
Rate of spread (m/min)	The linear rate of advance of a fire front in the direction perpendicular to the fire front	Potential Strengths: precise and continuous quantification of time interval between flaming front passage at two points. Weakness: while the fire cable allows for relatively prodigious spatial estimates compared to thermocouples, there are still spatial limitations as fire burns in three dimensions	Hand-held stop watch method is inexpensive but does not produce precision estimates necessary for physics based models. A series of thermocouples, while generally inexpensive, inherently result in spatial gaps that reduce temporal precision and requires numerous wires. Airborne remote sensing lacks the necessary temporal and spatial precision due to resolution limitations
Heat duration (time)	The length of time that heat occurs at a given point. Time at lethal heat (e.g., >60 °C) is often cited	Potential Strengths: given appropriate temperature calibration, greater temporal and spatial quantification as opposed to thermocouples Weakness: similar to thermocouples, temperature calibration is necessary; potential for heat capacity challenges; and cannot easily measure elevated temperatures (e.g., 10 cm above the soil surface)	Thermocouples are frequently used to measure surface heat duration with modest success

## Supplementary Materials

The following are available online at http://www.mdpi.com/1424-8220/16/10/1712/s1, Figures S1–S9: DTS data for plots 1–3 for each fiber (a) average temperature; (b) standard deviation of temperatures; (c) total thermal energy; and (d) maximum temperature vs. distance along fire cable; Figures S10–S12: DTS data from plots 1–3 copper fiber (a) maximum temperature (°C) recorded every 50.6 cm along fire cable; (b) Google Earth image showing location of burn plot; and (c) maximum temperature (°C) vs. approximate location; and Table S1: Total thermal energy (*TTE*) summed during burn at specific location along fire cable. Any additional data not covered by numerical information provided in this paper will be archived at the University of Massachusetts Amherst and may be requested from Christine E. Hatch (chatch@geo.umass.edu).
