# Supplementary material for: Use of Distributed Temperature Sensing Technology to Characterize Fire Behavior
Source: Sensors (Basel). 2016 Oct 17;16(10):1712. doi: 10.3390/s16101712 (PMC5087500; doi:10.3390/s16101712)
Supplement: Supplementary file 1 [file sensors-16-01712-s001.pdf]

Supplementary Materials: Use of Distributed Temperature Sensing Technology to Characterize Fire Behavior

Douglas Cram, Christine E. Hatch, Scott Tyler and Carlos Ochoa

1. Introduction

The following Supporting Information contains an additional table showing total thermal energy summed over three burns (Table S1, see Figure 7 in the manuscript). Each fiber's performance in plot 1–3 is graphed as follows: (a) average temperatures; (b) standard deviation of temperatures; (c) total thermal energy; and (d) maximum temperature vs. distance along fire cable (Figures S1–S9). DTS data from copper fiber in plots 1–3 is graphed showing (a) maximum temperature (°C) recorded every 50.6 cm along fire cable; (b) Google Earth image showing location of burn plot; and (c) maximum temperatures (°C) vs. approximate location in plot (Figures S10–S12).

Table S1. Total thermal energy (*TTE*) summed during burn at specific location along fire cable.

Location and Sensor	*TTE* (°C) [a] at 55 m [b]	*TTE* (°C) at 100 m	*TTE* (°C) at 123 m
**Plot 1**			
Thermocouple	33025	48185	46762
DTS fiber coating			
polyimide	37164	54070	37910
copper	49882	58698	48614
acrylate	52221	50751	38596
**Plot 2**			
Thermocouple	31287	43641	48201
DTS fiber coating			
polyimide	34935	40732	43659
copper	41614	51928	56611
acrylate	36872	43782	56611
**Plot 3**			
Thermocouple	30829	42795	47370
DTS fiber coating			
polyimide	45121	42899	76569
copper	49624	45713	55365
acrylate	41650	49514	40760

[a] Total thermal energy (*TTE*) is equal to the sum of temperatures (°C) measured during burn. *TTE* is normalized to the number of temperatures recorded and length of thermal wave (in seconds); [b] Meter mark along fiber-optic cable nearest thermocouple where total thermal energy calculation was measured.

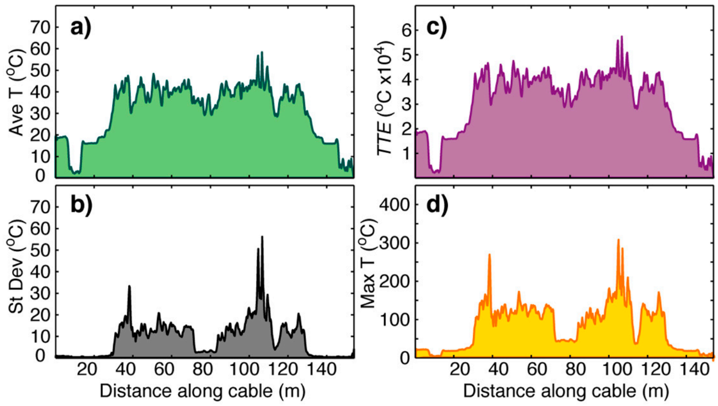

Figure S1. Plot 1 polyimide coated fiber: (**a**) Average temperature; (**b**) Standard deviation of temperature; (**c**) Total thermal energy during burn; and (**d**) Maximum temperature vs. distance along fire cable.

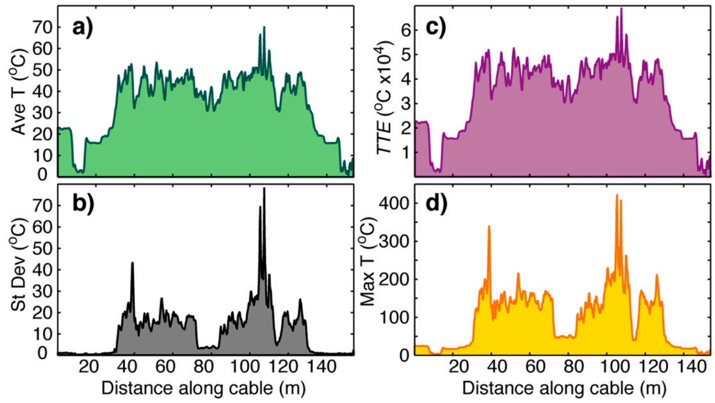

Figure S2. Plot 1 copper coated fiber: (**a**) Average temperature; (**b**) Standard deviation of temperatures; (**c**) Total thermal energy during burn; and (**d**) Maximum recorded temperature vs. distance along fire cable.

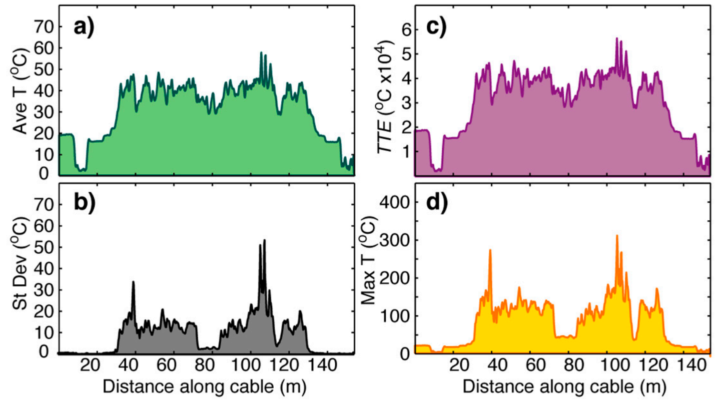

Figure S3. Plot 1 acrylate coated fiber: (**a**) Average temperature; (**b**) Standard deviation of temperatures; (**c**) Total thermal energy during burn; and (**d**) Maximum recorded temperature vs. distance along fire cable.

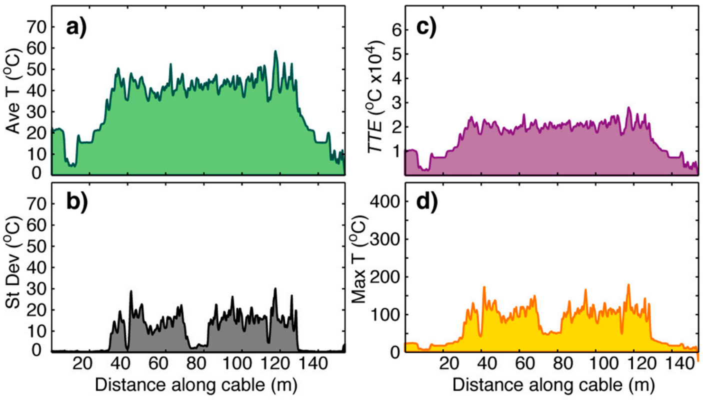

Figure S4. Plot 2 polyimide coated fiber: (**a**) Average temperature; (**b**) Standard deviation of temperatures; (**c**) Total thermal energy during burn; and (**d**) Maximum recorded temperature vs. distance along fire cable.

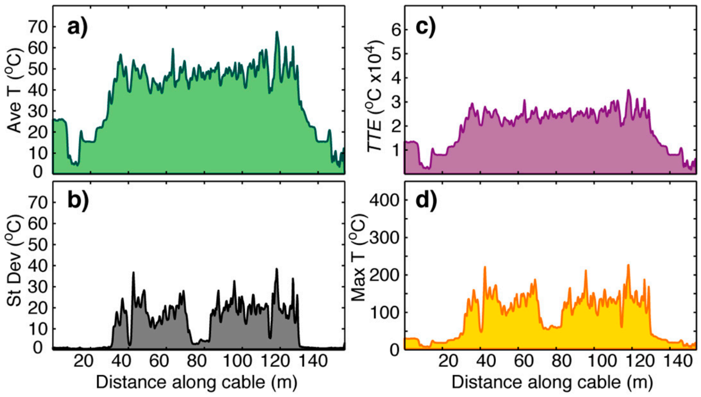

Figure S5. Plot 2 copper coated fiber: (**a**) Average temperature; (**b**) Standard deviation of temperatures; (**c**) Total thermal energy during burn; and (**d**) Maximum recorded temperature vs. distance along fire cable.

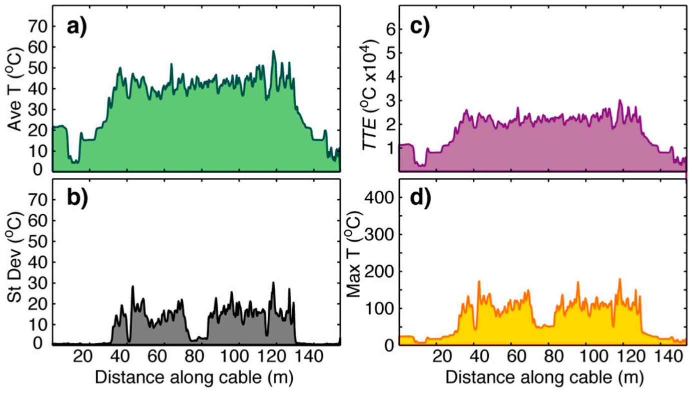

Figure S6. Plot 2 acrylate coated fiber: (**a**) Average temperature; (**b**) Standard deviation of temperatures; (**c**) Total thermal energy during burn; and (**d**) Maximum recorded temperature vs. distance along fire cable.

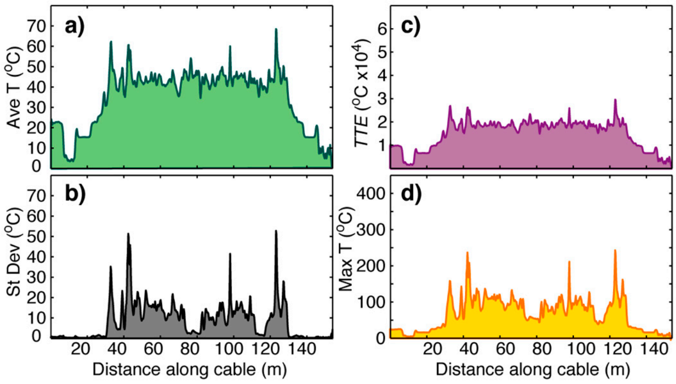

Figure S7. Plot 3 polyimide coated fiber: (**a**) Average temperature; (**b**) Standard deviation of temperatures; (**c**) Total thermal energy during burn; and (**d**) Maximum recorded temperature vs. distance along fire cable.

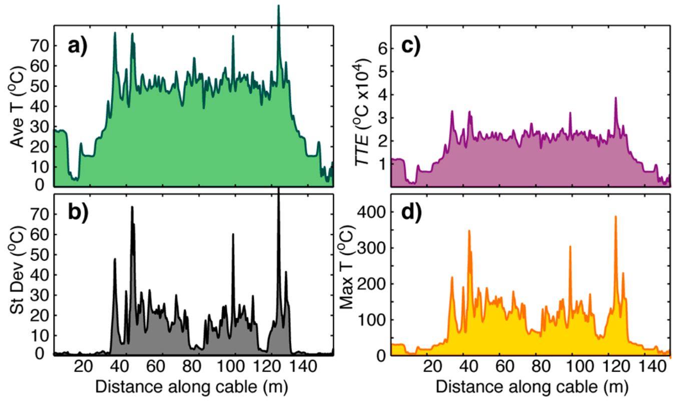

Figure S8. Plot 3 copper coated fiber: (**a**) Average temperature; (**b**) Standard deviation of temperatures; (**c**) Total thermal energy during burn; and (**d**) Maximum recorded temperature vs. distance along fire cable.

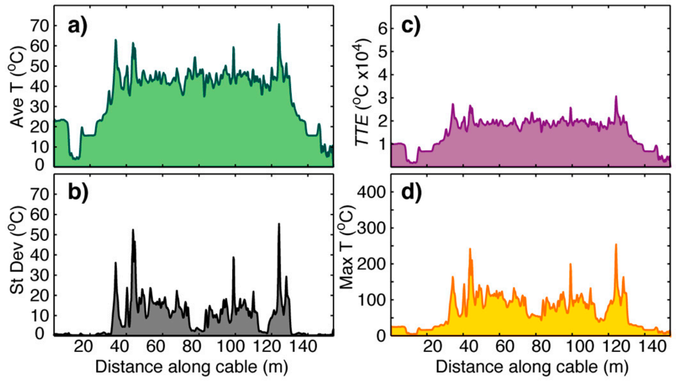

Figure S9. Plot 3 acrylate coated fiber: (**a**) Average temperature; (**b**) Standard deviation of temperatures; (**c**) Total thermal energy during burn; and (**d**) Maximum recorded temperature vs. distance along fire cable.

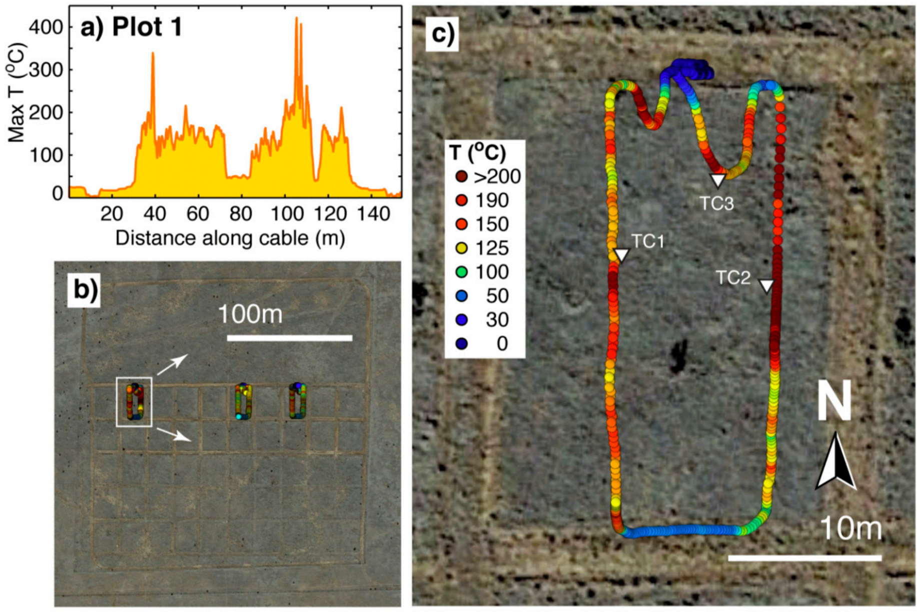

Figure S10. DTS data from plot 1 copper coated fiber: (**a**) Maximum temperature (°C) recorded every 50.6 cm along fire cable; (**b**) Google Earth image showing location of burn plot; and (**c**) Maximum temperatures (°C) vs. approximate location. Thermocouples (triangles) and individual data point locations are approximate. As described in Figure 3, burns began with a back fire (began in SE and burned toward the NE; at ~100 m and T2), continued with a flank fire (began in the NE and burned toward the W; at ~123 m and T1), and concluded with a head fire (began in the NW and burned toward the S; at ~55 m and T3). Note calibration locations outside the plot to the north, and sections of cable outside the plot not subjected to burning in the northeast and south.

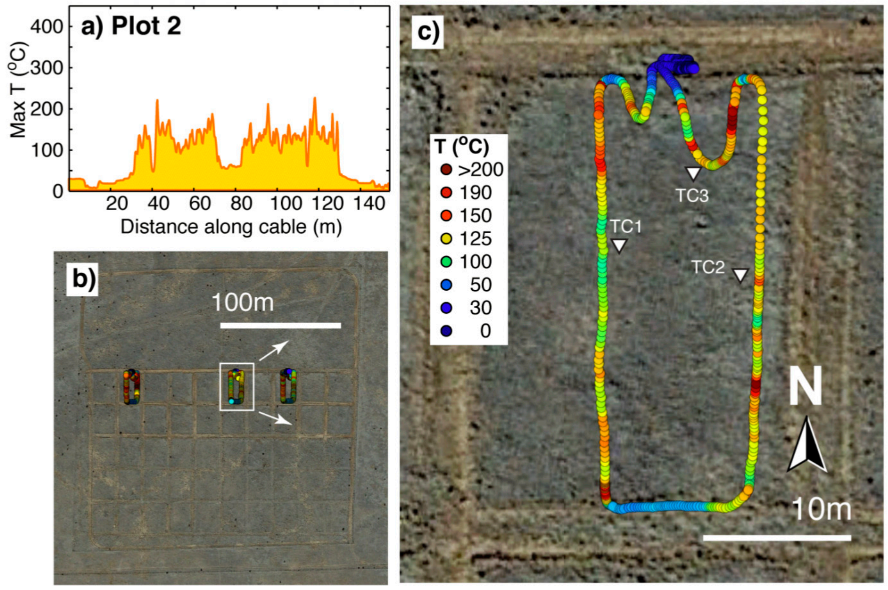

Figure S11. DTS data from plot 2 copper coated fiber: (**a**) Maximum temperature (°C) recorded every 50.6 cm along fire cable; (**b**) Google Earth image showing location of burn plot; and (**c**) Maximum temperatures (°C) vs. approximate location. Thermocouples (triangles) and individual data point locations are approximate. As described in Figure 3, burns began with a back fire (began in SE and burned toward the NE; at ~100 m and T2), continued with a flank fire (began in the NE and burned toward the W; at ~123 m and T1), and concluded with a head fire (began in the NW and burned toward the S; at ~55 m and T3). Note calibration locations outside the plot to the north, and sections of cable outside the plot not subjected to burning in the northeast and south.

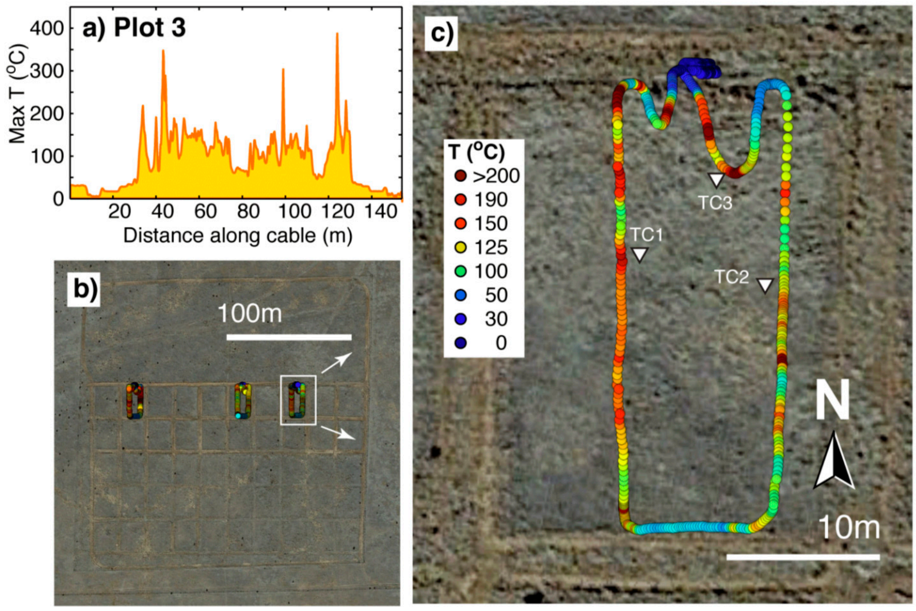

Figure S12. DTS data from plot 3 copper coated fiber: (**a**) Maximum temperature (°C) recorded every 50.6 cm along fire cable; (**b**) Google Earth image showing location of burn plot; and (**c**) Maximum temperatures (°C) vs. approximate location. Thermocouples (triangles) and individual data point locations are approximate. As described in Figure 3, burns began with a back fire (began in SE and burned toward the NE; at ~100 m and T2), continued with a flank fire (began in the NE and burned toward the W; at ~123 m and T1), and concluded with a head fire (began in the NW and burned toward the S; at ~55 m and T3). Note calibration locations outside the plot to the north, and sections of cable outside the plot not subjected to burning in the northeast and south.
